# A comparison of perceived uselessness between centenarians and non-centenarians in China

**DOI:** 10.1186/s12877-018-0944-7

**Published:** 2018-10-22

**Authors:** Yuan Zhao, Hong Fu, Aimei Guo, Li Qiu, Karen S. L. Cheung, Bei Wu, Daniela Jopp, Danan Gu

**Affiliations:** 1 Ginling College, School of Geographical Science, Nanjing Normal University, Nanjing, China; 2 Jiangsu Center for Collaborative Innovation in Geographical Information Resource Development and Application, Nanjing, China; 3 School of Psychology, Nanjing Normal University, Nanjing, China; 4 Ginling College, International Center for Aging Studies, Nanjing Normal University, Nanjing, China; 5 Independent Researcher, New York, USA; 6 Mindlink Institute, Hong Kong, Hong Kong; 7 Rory Meyers College of Nursing and NYU Aging Incubator, New York University, New York, USA; 8 Department of Psychology and National Centre for Research LIVES, University of Lausanne, Lausanne, Switzerland; 9 United Nations Population Division, Two UN Plaza, DC2-1910, New York, NY 10017 USA

**Keywords:** Centenarians, Self-perceived uselessness, China, Healthy longevity, CLHLS

## Abstract

**Background:**

Self-perceived uselessness is associated with poorer health in older adults. However, it is unclear whether there is a difference in self-perceived uselessness between centenarians and non-centenarians, and if so, which factors contributed to the difference.

**Methods:**

We used four waves of a nationwide longitudinal dataset from 2005 to 2014 in China to investigate these research goals. We first performed multinomial logit regression models to examine the risk of the high or moderate frequency of self-perceived uselessness relative to the low frequency among centenarians (5778 persons) in comparison with non-centenarians aged 65–99 (20,846 persons). We then conducted a cohort analysis for those born in 1906–1913, examining differences in self-perceived uselessness between those centenarians and those died between ages 91 and 99 during 2005–2014.

**Results:**

Compared to persons aged 65–79, centenarians had 84% (relative risk ratio (RRR) = 1.84, 95% CI:1.69–2.01) and 35% (RRR = 1.35, 95% CI: 1.25–1.46) higher risk to have the high frequency and the moderate frequency of feeling useless versus low frequency, respectively, when only demographic factors were controlled for. However, centenarians had 31% (RRR = 0.69, 95% CI: 0.54–0.88), 43% (RRR = 0.57, 95% CI: 0.49–0.68), and 25% (RRR = 0.75, 95% CI: 0.67–0.83) lower risk, respectively, to have the high frequency of self-perceived uselessness relative to the low frequency when a wide set of study covariates were controlled for. In the case of the moderate versus the low frequency of self-perceived uselessness, the corresponding figures were 18% (RRR = 0.82, 95% CI: 0.66–1.02), 22% (RRR = 0.78, 95%CI: 0.67–0.90), and 13% (RRR = 0.87, 95% CI: 0.79–0.96), respectively. The cohort analysis further indicates that those who became centenarians were 36–39% less likely than those died at ages 91–94 to report the high and the moderate frequencies of self-perceived uselessness versus the low frequency; no difference was found between centenarians and those died at ages 95–99. In both period and cohort analyses, behavioral and health-related factors affected the perception substantially.

**Conclusions:**

Overall, centenarians were less likely to perceive themselves as useless compared to non-centenarians of younger birth cohorts when a wide set of covariates were considered and non-centenarians of the same birth cohort. How centenarians manage to do so remains an open question. Our findings may help improve our understanding about the longevity secrets of centenarians.

## Background

Evidence from various populations has indicated that a strong sense of usefulness to others among older adults plays a crucial role in shaping positive views about their own aging, health behaviors and adequate adaptations that contribute positively to their good health and psychological wellbeing, and even longevity [1–15]. By contrast, perceiving one’s life as useless is associated with higher prevalence of chronic diseases [16, 17], poorer cognitive function and mental health status [7, 18–20], poorer self-rated health and life satisfaction [7, 21–24], lower physical functioning [5–7, 11, 25], and higher risk of mortality [6, 8, 10, 15, 26–29]. Perceptions of uselessness are also linked with higher levels of depression and lower levels of social and physical activity engagements, self-efficacy and self-esteem [5, 6, 11]. Although these studies have enriched our understanding about the associations between self-perceived uselessness and health behaviors, psychological wellbeing, and health outcomes [7, 30], most of these studies focused on general populations of older adults. It is less clear whether low levels of perception of uselessness still play a crucial role at very old age in helping long-lived persons reach successful aging and healthy longevity. Given the statistical robustness of self-perceived uselessness in affecting behaviors and in predicting health outcomes in the existing literature [7, 15, 30], it thus may have important implications for public health interventions to study self-perception of uselessness among centenarians and compare it with that of non-centenarian older adults.

Centenarians are often considered as the best age group to study healthy longevity and successful aging [31, 32]. Although centenarians show a poorer physical health and cognitive function compared to younger older adults [31–35], their psychological wellbeing may not be in disadvantage [32, 36]. More importantly, evidence shows that in comparison with their same cohort peers, centenarians are more psychologically resilient and have higher levels of physical and cognitive function than those who died younger [32, 36, 37].

Researchers generally agree that there is a large variation in disease conditions, physical/cognitive functions, and psychological wellbeing among these long-lived individuals [31, 35, 38], and that centenarians may follow a different trajectory of health decline from those non-centenarians [32]. Thus, the significance of comparison of self-perception of uselessness between centenarians and non-centenarians cannot be undervalued. Yet, studies about centenarians’ own views on aging or self-perceived usefulness are virtually nonexistent in the literature. Do centenarians have a more positive perception about their usefulness than their younger counterparts? And if so, what are the factors that could explain the differences? To our knowledge, there is currently no study available to address these research questions, possibly due to unavailability of data. This present study thus aims to investigate these research questions using the largest centenarian sample in the contemporary world from a nationwide longitudinal survey in mainland China.

## Methods

### Study sample

The four latest waves of datasets conducted in 2005, 2008/2009 (thereafter as 2008), 2011/2012 (thereafter as 2011), and 2014 from the Chinese Longitudinal Healthy Longevity Survey (CLHLS) were used to address our research questions. These four waves of datasets were pooled together to obtain more robust results. The 1998, 2000, and 2002 waves were excluded from our analyses in that some key variables used in the analyses were not available in these first three waves. The CLHLS is an ongoing project. Its samples were randomly selected from the half of the counties/cities in 22 of 31 provinces in mainland China. The proportion of the population of the sampled 22 provinces was about 87% of China in 2010 [7, 8]. The remaining nine provinces were excluded in the CLHLS to avoid age-reporting inaccuracy at oldest-old ages among non-Han minorities [39]. In 2008 and later waves, the CLHLS included an additional county (Chenmai County) with relatively good quality of age reporting quality from Hainan Province, one of these nine provinces. The present study includes 26,624 participants with 48,476 observations in 2005–2014. Among the 26,624 participants, 5778 were centenarians at the time of interview. The detailed sampling procedures and the assessments of data quality of the CLHLS can be found elsewhere and thus are not described here [7, 39].

One of the biggest challenges for centenarian studies is to validate the participants’ age for countries where a vital registration system is not well-developed, including China [39]. The CLHLS collected data on the date of birth and age. If the age of a respondent was reported in the lunisolar calendar, the age was converted into the solar calendar. The validation of respondents’ (especially centenarians’) self-reported ages in the CLHLS involved several rigorous procedures with different sources [39, 40]. These sources included household registration data, date of birth certification, genealogical data if available, marriage certificate if available, birth history, sibling history, and any other records available that could be used for validation [39, 40]. The systematic assessments showed that the accuracy of age reporting was high [39].

### Measurements

#### Self-perceived uselessness

Self-perceived uselessness was measured in the CLHLS by a single question: “As you age, do you feel more useless?” This question was designed based on an item from the Attitude toward Own Aging subscale of the Philadelphia Geriatrics Center Morale Scale [11, 12]. There were six response categories: always, often, sometimes, seldom, almost never or never, and unable to answer. Following previous research [5, 15, 30], we combined “always” and “often” into one category, and “seldom” and “almost never or never” into one category. We then renamed the new categories into *high frequency*, *moderate frequency*, *low frequency*, and unable to answer the question. The purpose of keeping the category “unable to answer” is to have the original information as intact as possible, whereas the purpose of collapsing five categories into three is to obtain more robust results because the sample size of some categories was relatively small. Of the 6207 participants who selected “unable to answer,” around 90% of them indicated that their refusal was due to poor health [5]. Although “unable to answer” was modeled, its results were not presented in the main text for a better focus on research objectives and enhanced presentation. These results are available upon request.

#### Associated factors

One recent study proposed a framework highlighting factors associated with self-perceived useless [30]. The framework, named REHAB, includes resource (R), social environments (E), health (H), demographic attributes (A), and behaviors (B). We relied on this framework to investigate possible factors that affect the difference in self-perceived uselessness between centenarians and non-centenarians. Specifically, we selected few major factors in each abovementioned domain. Demographic attributes included sex (men vs. women), urban-rural residence (urban vs. rural), and ethnicity (Han vs. non-Han). Resource factors consisted of years of schooling (0, 1–6, and 7+), financial independence (yes vs. no), lifetime primary occupation (white-collar occupation vs. others), and whether a participant was covered by a state medical insurance program (yes vs. no). If the respondent had a retirement wage or a pension, he or she was considered as financially independent. The state medical insurance programs included the new rural cooperative medical scheme, urban resident medical scheme, and urban employee medical scheme [41]. The respondents were asked to provide information whether they were covered by any of these three schemes (yes vs. no). Social environmental factors included current marital status (currently married vs. no), co-residence with children (yes vs. no), providing monetary support to children (yes vs. no), receiving monetary support from children (yes vs. no). Behavioral factors were measured by frequency of leisure activities. Levels of leisure activities were represented by the sum of frequencies of six items, including doing housework, gardening, raising domestic animals or poultry, reading books/newspapers, watching TV/listening to radio, and any other personal outdoor activities. For each item, response category used a five-point Likert-scale from never or almost never (score = 0) to almost daily (score = 4). The reliability coefficient of these six items is 0.66. We split the sample into three levels (low, moderate, and high) of activity engagement on the basis of the sample distribution.

Health status is possibly among the most important elements in affecting self-perception about aging [27]. In this study, we included two functional health measures: basic activities of daily living (ADL) and cognitive functioning. Although those with severe cognitive impairment were unlikely to answer the questions, evidence shows that those with slight or mild cognitive impairment still can answer easy self-report questions [42]. Following the common practice in the field [8], if a respondent needed assistance in performing any one of the six tasks (bathing, dressing, indoor transferring, toileting, eating, and continence), he or she was classified as ADL disabled; otherwise, he or she was considered as having no ADL disability. Cognitive impairment was measured by a validated Chinese version of the Mini-Mental State Examination (MMSE), which included six domains of cognition (i. e., orientation, reaction, calculation, short memory, naming, and language) with a total score of 30 [43]. A respondent was considered as cognitively impaired if his or her MMSE score was less than 24; otherwise, he or she was considered as cognitively normal [43]. An alternative cut-point score of 18 was also examined and yielded very similar results. The selection of factors and our coding of the variables were consistent with one recent study [44].

#### Analytical strategy

Because the self-perceived uselessness item in the current study was classified into four response categories (i.e., high frequency, moderate frequency, low frequency, and unable to answer), multinomial logistic regression models were employed to investigate whether centenarians were less likely to perceive themselves as useless than non-centenarians, and if so, which factors were associated with high or moderate frequency of self-perceived uselessness relative to the low frequency (the reference group). The results were reported in relative risk ratios (RRRs), which indicate how the risk of the high or moderate frequency of self-perceived uselessness relative to the low frequency in centenarians compared to the risk of the high or moderate frequency of self-perceived uselessness relative to the low frequency in non-centenarians. An RRR > 1 indicates that centenarians are more like to have the high or moderate frequency of self-perceived uselessness rather than to have the low frequency in comparison with non-centenarians, and vice versa. Some alternative approaches were also tested, including generalized ordered logistic regression models and binary logistic regression models. The generalized ordered logit models treated the self-perceived uselessness as an ordinal variable, whereas the logistic regression models treated the self-perceived uselessness as a dichotomous variable. Both sets of regression models produced very similar results. Similar to previous studies [15, 30], we pooled all four waves of the data together and adjusted for intrapersonal correlation across waves to obtain more robust and reliable results in that some respondents (both centenarians and non-centenarians) had more than two observations during the study period 2005–2014.

Two sets of analysis were designed: Period and cohort. The period analysis relied on the pooled dataset of the latest four waves from 26,624 respondents with 48,476 observations. Six nested models were analyzed for period analysis with different sets of control variables. Model I included demographic attributes (i.e., age, sex, urban-rural residence, ethnicity); Model II included resource factors (educational attainment, primary life occupation, economic independence, family economic condition, and access to healthcare services when in need) plus demographic attributes; Model III included social environmental factors (marital status, co-residence with children, primary caregivers) in addition to demographic attributes; Model IV included behavioral factors in terms of participation in leisure activities plus demographic attributes; Model V included health variables measured by ADL disability and cognitive impairment in addition to demographic attributes; and Model VI controlled for all covariates used in Models I to V. A variable reflecting the survey year was also included in all models to account for possible variations over time.

The same modeling strategy was applied to a cohort analysis that focused on those 2921 individuals who were born in 1906–1913. The cohort analysis compared two groups of people who were all born in the 1906–1913: one group survived to age 100 and the other group who reached age 90 yet died before age 100 in the study period 2005–2014. The reason that we selected these birth cohorts was because individuals who were born in 1906–1913 would all have passed age 100 in the 2014 wave if they were still alive so that we could identify for each respondent whether he or she lived to age 100 or not. An indicator variable capturing whether each respondent survived to age 100 or not was included in the model. This indicator variable had three categories: died at ages 91–94, died at ages 95–99, and died at age 100 or beyond. A variable of single year of birth cohort was included, while the variables of age and wave were dropped in all models of the cohort analysis.

Among those samples included in the analysis, the proportions of missing values for all study variables were less than 2%. To keep as many cases as possible in the analyses, we imputed these missing values using a regression-based approach by assuming that those respondents with missing values would have the same answer as those without missing values if their demographics, resources, social environments, behaviors, and health were the same. We also used other approaches (such as the mode for categorical variables and means for continuous variables) to impute the missing values and produced very similar results. In regression analyses, we did not apply the sampling weights because the weighted regression results could unnecessarily enlarge standard errors when the variables that are used for a construction of sampling weight are controlled for in the regression models [7, 8, 45]. All analyses were performed using Stata version 15.

## Results

Table 1 presents the percentage distribution of study variables by the frequency of self-perceived perception of uselessness for 48,476 observations collected in 2005–2014 from 26,624 participants. A smaller proportion of the centenarians reported high frequency of self-perceived uselessness compared to non-centenarians, yet centenarians had a much higher proportion of “unable to answer”. Among the 2921 individuals who were born in 1906–1913 included in the cohort analysis, those who became centenarians in 2005–2014 had a higher proportion of the low frequency of self-perceived uselessness compared to those who died between ages 91 and 99 in 2005–2014.

**Table 1:** Distribution of the pooled datasets: 2005, 2008, 2011, and 2014 waves of the CLHLS

Variables	Sample % ^a^	Self-perceived uselessness (percentage)			
		Always/often	Sometimes	Seldom /never	Unable to answer
Total observations	48,476 (100%)	11,147 (23.0%) ^b^	15,122 (31.2%) ^b^	16,000 (33.0%)^b^	6207 (12.8%)^b^
Age groups					
Age 65–79	30.3	19.7	34.3	43.8	2.3
Age 80–89	26.7	26.4	33.3	32.7	7.6
Age 90–99	26.0	24.7	29.9	28.1	17.3
Age 100+	17.0	20.9	24.4	21.9	32.8
Other demographics					
Female	56.4	25.0	30.2	28.8	16.0
Male	43.6	20.4	32.5	38.4	8.7
Non-Han ethnicity	16.3	19.3	34.0	34.2	12.5
Han ethnicity	83.7	23.7	30.7	32.8	12.9
Resources					
Own education, 0 years of schooling	66.0	24.9	30.7	28.5	15.9
Own education, 1–6 years of schooling	25.1	20.8	32.9	38.7	7.6
Own education, 7+ years of schooling	8.9	15.0	29.9	50.2	4.9
Rural	56.4	24.7	31.4	30.2	13.8
Urban	43.6	20.9	30.9	36.7	11.6
Non-white collar occupation	92.3	23.7	31.3	31.7	13.3
White collar occupation	7.7	14.2	29.7	49.2	6.9
Economic dependence	72.2	25.2	30.7	28.4	15.7
Economic independence	27.8	17.3	32.5	45.0	5.2
Fair or poor family economic condition	84.8	24.1	31.5	31.0	13.4
Rich family economic condition	15.2	16.8	29.3	44.5	9.3
Not covered by state medical insurance scheme	8.5	35.7	27.7	15.5	21.1
Covered by state medical insurance scheme	91.5	21.8	31.5	34.7	12.0
Family/social support					
Currently not married	66.0	24.5	30.2	28.2	17.1
Currently married	34.0	20.1	33.1	42.3	4.5
Coresidence with children - no	39.3	24.3	32.5	35.6	7.6
Coresidence with children - yes	60.7	22.2	30.4	31.3	16.2
Receiving money/food from children - no	20.0	21.5	28.5	33.5	16.6
Receiving money/food from children - yes	80.0	23.4	31.9	32.9	11.9
Giving money/food to children - yes	77.0	24.5	30.2	30.2	15.1
Giving money/food to children - no	23.0	18.1	34.5	42.5	5.0
Behaviors					
Frequency of leisure activities-low level	75.6	25.2	30.6	28.5	15.7
Frequency of leisure activities- medium level	11.0	16.7	34.6	43.4	5.4
Frequency of leisure activities -high level	13.6	15.8	31.6	49.8	2.8
Health conditions					
ADL independent	74.6	21.8	33.7	38.1	6.5
ADL dependent	25.4	26.4	24.0	18.2	31.5
Cognitively unimpaired	60.1	21.1	35.7	41.7	1.4
Cognitively impaired	39.9	25.8	24.4	19.9	30.0
Survey years					
Wave 2005	31.6	23.2	32.2	33.4	11.3
Wave 2008	33.7	23.9	29.1	31.9	15.1
Wave 2011	20.0	22.1	31.9	34.4	11.6
Wave 2014	14.7	21.7	33.0	33.0	12.3
Cohorts born in 1906–1913 (2921) ^c^					
Died at ages 91–94	10.2	29.4	33.1	21.7	15.7
Died at ages 95–99	58.8	23.5	28.0	27.1	21.4
Died at ages 100+	31.0	25.3	29.3	29.3	16.1

Note: (1) Except for the total number of observations in the top line, all numbers were percentages unless otherwise stated. (2) a, this column referred to percentage distribution of each category of the study variables among 48,476 observations from 26,624 individuals who were interviewed from 2005 to 2014. The distributions by 26,624 individuals at their baseline were similar to what were presented in the Table 3b, percentages of self-perceived uselessness were calculated by row. The row sum of percentage of self-perceived uselessness may not be equal to 100% due to roundness. (4) c, Distribution for cohorts born in 1906–1913 was based on 2972 individuals who were followed-up till the 2014 wave or died before 2014. Those who were lost to follow-up between 2005 and 2014 were excluded. (5) All distributions were unweighted

Table 2 presents the relative risk ratios (RRRs) of reporting high and moderate frequencies versus the low frequency of self-perceived uselessness for centenarians as compared to older adults in other age groups from the period analysis. Model I reveals that although centenarians had no difference in self-perceived uselessness compared to octogenarians and nonagenarians when only demographics were controlled for, they were respectively associated with 84% (RRR = 1.84, 95% CI: 1.69–2.01) and 35% (RRR = 1.35, 95% CI: 1.25–1.46) higher risk of reporting the high and the moderate frequencies of self-perceived uselessness relative to the low frequency in comparison with older adults aged 65–79. Model II shows that the elevated RRRs were mildly attenuated to 52% (RRR = 1.52, 95% CI: 1.39–1.67) and 23% (RRR = 1.23, 95% CI: 1.13–1.33), respectively, when resources were added to Model I; and Model III shows that these RRRs were 56% (RRR = 1.56, 95% CI: 1.42–1.72) and 21% (RRR = 1.21, 95% CI: 1.11–1.31), respectively, when social environmental factors were included in Model I. So far, modeling results reveal that centenarians were more likely to perceive themselves as useless compared to the youngest older adults aged 65–79 and had no difference in self-perceived uselessness as compared to older adults aged 80–99.

**Table 2:** Relative risk ratios of the high and the moderate frequencies relative to the low frequency of self-perceived uselessness among centenarians in comparison with non-centenarians, CLHLS 2005–2014

Ages at survey	Model I	Model II	Model III	Model IV	Model V	Model VI
High frequency relative to low frequency						
Ages 100+ vs. ages 65–79	1.84^***^ (1.69–2.01)	1.52^***^ (1.39–1.67)	1.56^***^ (1.42–1.71)	1.08 (0.98–1.18)	0.97 (0.89–1.07)	0.69^**^ (0.54–0.88)
Ages 100+ vs. ages 80–89	1.04 (0.95–1.13)	0.99 (0.91–1.08)	0.99 (0.91–1.08)	0.75^***^ (0.69–0.82)	0.66^***^ (0.60–0.72)	0.57^***^ (0.49–0.68)
Ages 100+ vs. ages 90–99	0.98 (0.90–1.07)	0.96 (0.88–1.04)	0.98 (0.90–1.07)	0.85^*^ (0.78–0.93)	0.78^***^ (0.71–0.85)	0.75^***^ (0.67–0.83)
Moderate frequency relative to low frequency						
Ages 100+ vs. ages 65–79	1.35^***^ (1.25–1.46)	1.23^***^ (1.13–1.33)	1.21^***^ (1.11–1.31)	1.11^*^ (1.02–1.21)	1.09^*^ (1.00–1.19)	0.82+ (0.66–1.02)
Ages 100+ vs. ages 80–89	1.04 (0.96–1.13)	1.01 (0.93–1.10)	1.00 (0.92–1.08)	0.93+ (0.85–1.00)	0.89^**^ (0.82–0.96)	0.78^**^ (0.67–0.90)
Ages 100+ vs. ages 90–99	1.00 (0.92–1.09)	0.99 (0.91–1.07)	0.99 (0.92–1.08)	0.95 (0.88–1.03)	0.91^*^ (0.85–0.99)	0.87^**^ (0.79–0.96)

Note: (1) Figures in the table were relative risk ratios based on unweighted multinomial logistic regression models adjusting for intrapersonal correlation from 26,624 respondents consisting of 48,476 observations. (2) The high frequency of feelings of useless referred to always/often; the moderate frequency referred to sometimes; and the low frequency referred to seldom/never. The category of “unable to answer” of the self-perceived uselessness was included in the analyses, but their results were not presented because they are not our focuses. (3) Model I controlled for demographic attributes (sex, urban-rural residence, ethnicity), and the years of survey; Model II added resource factors (educational attainment, primary life occupation, economic independence, family economic condition, and adequate access to healthcare services when in need) in Model I; Model III controlled for social environmental factors (marital status, coresidence with children, primary caregivers) in addition to covariates in Model I; Model IV controlled for behavioral factors (leisure activities) in addition to covariates in Model I; Model V controlled for health-related factors (disability in activities of daily living and cognitive impairment) in addition to covariates in Model I; and Model VI controlled for all covariates in Models I to V. (4) + *p* < 0.1, ^*^p < 0.05, ^**^p < 0.01, ^***^p < 0.001

However, when behavioral factors were controlled in addition to demographics (Model IV), centenarians had 25% (RRR = 0.75, 95% CI: 0.69–0.82) and 15% (RRR = 0.85, 95% CI: 0.78–0.93) lower risk ratios of having the high frequency of self-perceived uselessness relative to the low frequency compared to octogenarians and nonagenarians, respectively, although they still had a 11% higher risk ratio of having the moderate frequency relative to low frequency of self-perception of uselessness than older adults aged 65–79. When ADL disability and cognitive impairment were controlled for in addition to demographics (Model V), the centenarians were 34% (RRR = 0.66, 95% CI: 0.60–0.72) and 22% (RRR = 0.78, 95% CI: 0.71–0.85) less likely than octogenarians and nonagenarians to have the high frequency of self-perceived uselessness relative to the low frequency. The corresponding figures for the moderate relative to the low frequency of self-perception were 11% (RRR = 0.89, 95% CI: 0.82–0.96) and 9% (RRR = 0.91, 95% CI: 0.85–0.99).

When all covariates were taken into consideration, centenarians had 31% (RRR = 0.69, 95% CI: 0.54–0.88), 43% (RRR = 0.57, 95%CI: 0.49–0.68), and 25% (RRR = 0.75, 95%CI: 0.67–0.83) respectively lower risk ratios of having the high frequency relative to the low frequency of self-perceived uselessness compared to older adults aged 65–79, 80–89, and 90–99. In the case of the moderate frequency relative to the low frequency, these reduced risk ratios were 18% (RRR = 0.82, 95% CI: 0.66–1.02), 22% (RRR = 0.78, 95% CI: 0.67–0.90), and 13% (RRR = 0.87, 95% CI: 0.79–0.96), respectively. These results of the sequential models indicate that all sets of factors played a certain role in distinguishing the self-perception of uselessness between centenarians and older adults at other age groups, but health practice and health conditions played a greater role than other factors.

In the case of the cohort analysis among those who were born in 1906–1913 and survived to age 91 or above, Table 3 shows that compared with those who died at ages 91–94, those who became centenarians were 36–39% less like to have the high and the moderate frequencies of self-perceived uselessness versus the low frequency when demographic factors plus either resources factors or social environmental factors were taken into account. When the participation in leisure activities or the health condition was taken into consideration, the relative risk of the high frequency versus the low frequency of self-perceived uselessness among centenarians was not statistically significant compared with those who died at age 91–94, although the RRR was significant in the case of the moderate frequency versus the low frequency controlling for health behaviors and demographics (Model IV). In other words, when participation in leisure activities and health condition of those who died at age 100 or beyond was the same as those who died at ages 91–94, there would have no difference in self-perceived uselessness between these two groups. In our sample, those who died at age 100 or beyond were in better health and were more likely to participation in leisure activities (not shown). When all factors were controlled for, there was only a very slight change in RRRs (Model VI) compared to the model that controlled for health (Model V). These results suggest that all factors under study other than participation in leisure activities and health conditions had a little impact on differentiating self-perception of uselessness between centenarians and those died at ages 91–94. This implies that participation in leisure activities and health condition played a greater role than other factors in differing those who lived to age 100 from those from the same birth cohort who died earlier in 2005–2014. No significant difference was found between those who died at age 100 or older and those who died at ages 95–99.

**Table 3:** Relative risk ratios of high and moderate frequencies relative to low frequency of self-perceived uselessness for those lived to age 100 and above in comparison with those lived to age 91 yet died before age 100 among those born in 1906–1913, CLHLS 2005–2014

Age at death	Model I	Model II	Model III	Model IV	Model V	Model VI
High frequency relative to low frequency						
Survived to age 100 vs. died at ages 91–94	0.62^*^ (0.41–0.91)	0.64^*^ (0.42–0.97)	0.62^*^ (0.41–0.84)	0.74 (0.48–1.12)	0.80 (0.52–1.22)	0.85 (0.55–1.32)
Survived to age 100 vs. died at ages 95–99	0.98 (0.77–1.24)	1.02 (0.80–1.29)	0.98 (0.78–1.24)	1.11 (0.88–1.43)	1.16 (0.91–1.47)	1.25 (0.97–1.60)
Moderate frequency relative to low frequency						
Survived to age 100 vs. died at ages 91–94	0.61^*^ (0.41–0.91)	0.62^*^ (0.42–0.93)	0.61^*^ (0.41–0.91)	0.64^*^ (0.43–0.97)	0.69+ (0.47–1.04)	0.71+ (0.48–1.06)
Survived to age 100 vs. died at ages 95–99	0.93 (0.75–1.17)	0.95 (0.76–1.19)	0.94 (0.75–1.17)	0.98 (0.78–1.23)	1.02 (0.81–1.28)	1.04 (0.82–1.31)

Note: (1) Figures in the table were relative risk ratios based on unweighted multinomial logistic regression models from 2921 respondents who were born between January 1, 1906 and December 31, 1913. (2) The high frequency of feelings of useless referred to always/often; the moderate frequency referred to sometimes; and the low frequency referred to seldom/never. The category of “unable to answer” of the self-perceived uselessness was included in the analyses, but their results were not presented because they are not our focuses. (3) Model I controlled for demographic attributes (sex, urban-rural residence, ethnicity), and the years of survey; Model II added resource factors (educational attainment, primary life occupation, economic independence, family economic condition, and adequate access to healthcare services when in need) in Model I; Model III controlled for social environmental factors (marital status, coresidence with children, primary caregivers) in addition to covariates in Model I; Model IV controlled for behavioral factors (leisure activities) in addition to covariates in Model I; Model V controlled for health-related factors (disability in activities of daily living and cognitive impairment) in addition to covariates in Model I; and Model VI controlled for all covariates in Models I to V. (4) + p < 0.1, ^*^p < 0.05, ^**^p < 0.01, ^***^p < 0.001

## Discussion

Based on the 2005, 2008, 2011, and 2014 four waves of the Chinese Longitudinal Healthy Longevity Survey, the largest nationally representative survey of older adults in China, we examined whether centenarians were less likely to perceive themselves as useless compared to younger cohorts. In general, we found that when only demographic attributes, socioeconomic resources, and social environmental factors were taken into consideration, centenarians had a higher proportion of having a negative self-perception about their own usefulness than younger generations aged 65–79, although the centenarians had a similar proportion compared to octogenarians and nonagenarians. However, when behavioral factors or heath conditions were taken into account, centenarians were less likely to have a perception of self-perceived uselessness than octogenarians and nonagenarians; and when all factors under study were controlled for, centenarian were also less likely to have a negative perception about their usefulness compared to all four younger age groups under study. More importantly, we further found from the cohort analysis of those born in 1906–1913 that in comparison with those who were from the same cohort but died at ages 91–94, those who became centenarians were less likely to perceive themselves as useless when demographic attributes, resource factors, and social environmental factors were controlled for. Overall, these results convey a clear message that centenarians hold more positive attitudes and views about their aging than non-centenarians, and that these positive views may attribute to better chances for survival.

One important finding of the present study is that health in terms of physical and cognitive functions and behaviors played a more important role than other factors in distinguishing the self-perceive uselessness between centenarians and non-centenarian older adults. Health behaviors were measured by leisure activities, which is closely related physical function. This finding provides additional evidence to support the contribution of health and active lifestyle to self-perception about one’s views and attitudes toward aging or usefulness. Studies have shown that a good health condition is associated with a better self-perception about one’s usefulness to family and others [30]. Our findings also enrich the existing literature on factors associated with self-perceived usefulness and self-perception of aging [15] and the literature on centenarians’ positive psychological attributes [26].

Research has indicated that perceptions of usefulness or uselessness may impact one’s health psychologically, behaviorally, and physiologically [7, 14, 15]. Physiologically, having a strong feeling of usefulness could avoid a dysregulation of the central nervous system, neurotransmitters, and/or immune system for the onset and progression of disease, disability, and other manifestations of aging [46, 47]. Behaviorally, positive views and attitudes about one’s aging could maintain healthy lifestyles that promote health [5]. Psychologically, a strong sense or perception about own usefulness to others could avoid diminishment of beliefs about self-control and self-efficacy, help prevent social isolation, and improve resilience capacity to deal with negative views/thoughts and difficulties in daily life [5, 15, 48].

It is possible that with increasing age, some very old adults, especially centenarians have developed strategies to cope with challenges and changes in their environments so that the sense of perceived usefulness to family, others, and society is maintained [6, 32, 49–52]. Some previous studies have shown that a fairly large portion of centenarians are still in a good function [35], that vast majority of centenarians are quite independent in performing daily activities when they were at early 90s [38], and that they are more psychologically resilient than younger peers of the same birth cohort, or even as resilient as those younger birth cohorts [32, 50]. Overall, our findings are generally in accordance with previous findings that centenarians were more physically and psychologically robust than nonagenarians of their same birth cohorts in handling stress, depression, or other unfavorable conditions than their cohort peers [32, 36–38, 50]. The lower levels of self-perceived uselessness among the centenarians imply that positive attitudes and views about aging may be an important pathway to reach age 100.

Our finding that centenarians are less likely to feel useless to family and others compared to their cohort peers underscores the importance of maintaining positive self-perceptions with age at an individual level and suggests that it may never be too late to promote positive perspectives of aging [30]. In order to achieve exceptional longevity, it is thus recommended to promote positive views and attitudes among older people by building and maintaining adequate emotional capacity, neutralizing negative emotionality, developing resistance to counteract negative age-associated stressors, and nurturing positive views of health and life [53, 54]. Given recent evidence on the negative perceptions of aging among older adults in China [55], and eroding practice of filial piety and respecting for older adults due to rapid social and economic changes [7, 56, 57], promoting positive views about one’s own aging and creation of age-friendly environments are especially timely and needed so as to achieving successful aging [58].

While highlighting the strengths, we acknowledge the following limitations. First, the CLHLS only used a single item to collect data on self-perceived uselessness, which may not capture the multidimensionality of the concept of uselessness [15, 28]. Second, because the CLHLS is not designed to be a cohort study, the sample size at each individual age only consists of a couple of hundred participants, which may be not sufficient in follow-up wave due to high mortality among these participants, although the total sample size is relatively large. Furthermore, the follow-up length only lasted for 10 years in the present study, which is a relatively short follow-up period. In addition, in our cohort analysis, more than 60% of the samples were aged 95–99 at the time of their initial interview and more than 85% of those who did not live to age 100 died at age 95–99. In other words, our cohort results mainly refer to a comparison between those aged to 95–99 years old and those who became centenarians. Overall, larger sample size and longer follow-up period are warranted to have more robust results. Third, although we examined factors associated with self-perceived uselessness for centenarians in comparison with non-centenarians and found that centenarians were more likely to have a low frequency of self-perceived uselessness compared with their same birth cohort peers who did not become centenarians. However, the casual mechanism responsible for how such positive attitudes or perceptions have enhanced their healthy longevity deserves further investigations. Studies combining phenotypic and genotypic data and adopting an interdisciplinary perspective may be a promising area for further explorations. Fourth, studies have shown that people may change their perception of age over time [59]. It is thus possible that the sense of longevity may influence their perceived usefulness, either negatively or positively, when they live longer and longer. Yet it is unknown whether, how, and to what extent aging itself or longevity can improve positive perception about own usefulness. We welcome more studies to shed light onto better understanding of the underlying mechanism of positive perception and longevity. Finally, although to our knowledge the present study is the first to investigate centenarians’ own perceptions about their usefulness in comparison with those of non-centenarians, many factors that moderate or mediate the association between self-perception of usefulness, and other factors such as psychosocial and biological traits that are important factors in linking self-perceptions of aging with longevity were not included in the analyses [60]. We hope that in the future more studies will investigate the underlying mechanism between self-perceptions of own aging and longevity.

## Conclusions

Based on a large nationally representative multi-wave survey of older adults in China from 2005 to 2014, we found that centenarians were less likely to have a negative perception about their own usefulness compared with younger elders when holding other personal characteristics and conditions equal and compared with those non-centenarian peers of their own birth cohorts. Our findings provided additional evidence to support the notion that centenarians are more positive about their aging. The results from this study could broaden our understanding of how internalized perceptions of aging could have significant consequences for the survival and longevity of older adults in a rapidly aging society.

## Abbreviations

ADL: Activities of daily living
CLHLS: Chinese Longitudinal Healthy Longevity Survey
MMSE: Mini-mental status examination
RRR: Relative Risk Ratio
